# Effect of Phosphate Binders and a Dietary Iron Supplement on the Pharmacokinetics of a Single Dose of Vadadustat in Healthy Adults

**DOI:** 10.1002/cpdd.1033

**Published:** 2022-02-16

**Authors:** Susan K. Paulson, Jimena Martinez, Rishikesh Sawant, Steven K. Burke, Ajit Chavan

**Affiliations:** ^1^ Akebia Therapeutics, Inc. Cambridge Massachusetts USA

**Keywords:** clinical trials, drug‐drug interactions, drug metabolism, nephrology, pharmacokinetics, renal disease

## Abstract

Vadadustat is a hypoxia‐inducible factor prolyl‐hydroxylase inhibitor being developed for the treatment of anemia in patients with chronic kidney disease. Sequelae of chronic kidney disease include hyperphosphatemia and anemia, which are frequently treated with phosphate binders and iron supplements, respectively. Two studies evaluating the pharmacokinetics, safety, and tolerability of a single oral dose of vadadustat coadministered with a phosphate binder or iron supplement were conducted in healthy adult participants. In study 1, 54 healthy women and men were administered vadadustat (300 mg) alone and 1 hour before, concurrently with, or 2 hours after a phosphate binder (sevelamer carbonate 1600 mg, calcium acetate 1334 mg, or ferric citrate 2000 mg). In study 2, 10 healthy men were administered vadadustat (450 mg) alone and concomitantly with the oral iron supplement ferrous sulfate (325 mg [equivalent to 65 mg of elemental iron]). Vadadustat exposure was reduced by coadministration with sevelamer carbonate, calcium acetate, ferric citrate, or ferrous sulfate. Geometric least squares mean ratios for area under the concentration‐time curve from time 0 to infinity were reduced 37% to 55% by phosphate binders and 46% by ferrous sulfate. However, when vadadustat was administered 1 hour before phosphate binders, 90% confidence intervals for vadadustat exposure were within the no‐effect boundaries of +50% to –33%, indicating that drug‐drug interactions can be reduced by administering vadadustat 1 hour before phosphate binders. Vadadustat was well tolerated when administered in conjunction with phosphate binders or an iron supplement.

Chronic kidney disease (CKD) is a major health burden, with a global prevalence of 697.5 million (9.1%) in 2017.^1^ Patients with CKD experience a range of disease‐related complications and have an increased risk for cardiovascular disease, end‐stage renal disease, infection, and mortality.^2^

Hyperphosphatemia and anemia are common complications of CKD that become increasingly prevalent as disease severity increases.^2^, ^3^ As CKD progresses, calcium‐phosphorus homeostasis becomes increasingly dysregulated, leading to impaired phosphorus excretion resulting in hyperphosphatemia, which has been linked to an increased risk of cardiovascular disease, kidney failure, and mortality in patients with CKD.^3^

Management of hyperphosphatemia generally involves using an oral phosphate binder to reduce phosphate absorption in combination with dietary interventions to decrease phosphorus intake, thereby lowering serum phosphate levels toward the normal range.^4^ Several types of phosphate binders can be prescribed, including calcium‐ and non–calcium‐based binding agents such as aluminum‐, polymer‐, and iron‐based agents.^3^

Anemia is another common complication of CKD that contributes to symptom burden and may arise because of iron or erythropoietin deficiency, blood loss, or increased erythrocyte turnover.^5^ Anemia in patients with CKD is managed by oral or intravenous iron supplementation and erythropoiesis‐stimulating agents (ESAs).^5^, ^6^

Vadadustat, an orally bioavailable small‐molecule inhibitor of hypoxia‐inducible factor prolyl‐hydroxylases (HIF‐PHIs), is in development for the treatment of anemia in both dialysis‐dependent and non–dialysis‐dependent patients with CKD. HIF‐PHIs are a possible alternative to the current standard of care for patients with CKD and anemia since ESA use is limited by an increased risk of cardiovascular events.^7^, ^8^, ^9^, ^10^, ^11^ By regulating HIF activity, vadadustat stimulates erythropoietin production and modulates iron metabolism.^12^, ^13^ Vadadustat has been shown to increase and maintain hemoglobin levels in patients with CKD who are or are not dialysis dependent and in patients who are treatment naive or converting from ESA therapy.^14^, ^15^ Vadadustat also has been shown to be well tolerated in healthy volunteers and patients with CKD.^13^, ^16^, ^17^

Vadadustat demonstrates dose‐proportional pharmacokinetics (PK), with maximum plasma concentrations reached ≈1 to 4 hours after oral administration. Vadadustat undergoes extensive metabolism, primarily via O‐glucuronidation to the inactive metabolite vadadustat‐O‐glucuronide before being eliminated in the urine and feces. The terminal elimination half‐life (t_½_) of vadadustat is 4.7 hours in healthy adults but ranges from 7.9 to 9.1 hours in patients with non–dialysis‐ and dialysis‐dependent CKD, respectively.^18^ Among patients with mild or moderate hepatic impairment, single oral administration of vadadustat has been shown to be well tolerated, with no clinically meaningful effect on systemic exposure.^19^

As hyperphosphatemia and anemia are common complications of CKD, phosphate binders and oral iron supplements are anticipated to be used concomitantly with vadadustat in a clinical setting, suggesting that there is a potential for drug‐drug interactions (DDIs). In particular, by forming a chelation complex with these agents, vadadustat absorption from the gastrointestinal tract may be altered.

Two phase 1 studies were conducted to determine whether a DDI exists between vadadustat and phosphate binders and ferrous sulfate. The first study assessed the effect of a single oral dose of a polymer‐based (sevelamer carbonate [Renvela]), calcium‐based (calcium acetate), or iron‐based (branded ferric citrate [Auryxia]) phosphate binder on the plasma PK of vadadustat. A second study assessed the effect of concurrent administration of a single dose of ferrous sulfate on the plasma PK of vadadustat and the inactive primary metabolite vadadustat‐O‐glucuronide. Both studies also aimed to assess the safety and tolerability of vadadustat coadministered with phosphate binders or supplemental iron.

## Materials and Methods

Two open‐label, phase 1 clinical pharmacology studies were conducted in healthy volunteers in accordance with the International Conference on Harmonisation Good Clinical Practice guidelines, US Food and Drug Administration regulations, and the principles of the Declaration of Helsinki. Study protocols were approved by institutional review boards for each study site at Advarra Institutional Review Board (Ontario, Canada) and IntegReview (Austin, Texas). All participants provided written informed consent before enrollment. Both studies were conducted at a single site; study 1 was conducted at Syneos Health Clinique Inc. (Quebec City, Quebec, Canada), and study 2 was conducted at Jasper Clinic (Kalamazoo, Michigan). Each study was prospectively registered on ClinicalTrials.gov (ClinicalTrials.gov identifiers NCT04299633 and NCT02327546) before enrolling its first participant.

### Study 1 Design

Study 1 was an open‐label, 3‐part, fixed‐sequence study to evaluate the effect of a single oral dose of a phosphate binder on the PK of a single dose of vadadustat. In each part, a single dose of vadadustat (2 × 150 mg oral tablets [300 mg total]) was administered alone and in combination with a single dose of 1 of 3 phosphate binders: sevelamer carbonate (2 × 800 mg oral tablets [1600 mg total]) in part 1; calcium acetate (2 × 667 mg oral gel capsules [1334 mg total]) in part 2; or ferric citrate (2 × 1 g tablets [2000 mg total]) in part 3. To investigate the effect of timing of administration of each study drug, each phosphate binder was administered in combination with vadadustat on 3 separate occasions, with a 1‐day washout period following the day of administration.

On day 1, vadadustat was administered immediately after a normocaloric breakfast totaling 665 kilocalories that included 1 individual cereal box, white bread, cheese, butter, sugar packet, milk with 2% milk fat, and 1 box of grape juice. On day 3, vadadustat and the phosphate binder were administered simultaneously immediately following breakfast. On day 5, vadadustat was administered after an overnight fast, and the phosphate binder was administered 1 hour later, followed immediately by breakfast. On day 7, the phosphate binder was administered immediately after breakfast, and vadadustat was administered 2 hours later. Participants were discharged from the study site on day 9, following collection of the final PK blood sample.

Study 1 enrolled healthy women and men aged 18 to 55 years, with a body mass index of 18.0 to 30.0 kg/m^2^ and a minimum body weight of 45 kg for women and 50 kg for men. Participants were excluded if they had a current or past history of cardiovascular, respiratory, gastrointestinal, hematologic, renal, hepatic, immunologic, metabolic, urologic, neurologic, dermatologic, psychiatric, or other major disease, or clinically significant history of hypercalcemia, iron overload, liver disease, hyperphosphatemia, ulcerative colitis, or gastrointestinal bleeding. Other key exclusion criteria included the use of any prescription medicine, over‐the‐counter multivitamin supplement, or nonprescription product (excluding acetaminophen ≤2 g/d) within 14 days before day –1; use of any cytochrome P450 or P‐glycoprotein inducers or inhibitors within 14 days prior to day –1; daily use of nicotine‐containing products within 6 months of screening; or consumption of any food or drink containing grapefruit juice, apple or orange juice, Seville or blood oranges, or vegetables from the mustard‐green family within 7 days before study drug administration. See Table S1 for detailed inclusion and exclusion criteria.

### Study 2 Design

Study 2 was an open‐label, randomized, single‐dose study to evaluate the effect of ferrous sulfate on vadadustat PK. Participants were admitted to the study site ≥20 hours before study drug administration. Participants received a single dose of vadadustat (3 × 150 mg oral tablets; 450 mg total) alone and in combination with a single dose of ferrous sulfate (325‐mg oral tablet; equivalent to 65 mg of elemental iron) in a randomized crossover fashion. The study used a 2‐period randomized crossover design in which each participant served as their own control. Study drugs were administered with water (8 oz [≈240 mL]) following an overnight fast (10 hours). Fasting continued for 4 hours after dosing, and water was restricted for 1 hour before and after dosing. Participants were seated during study drug administration and for 2 hours after dosing. There was a washout period of 4 days between each administration of the study drug, and participants remained at the study site until collection of the final PK blood sample.

Enrolled participants were healthy adult men aged 18 to 55 years with a body mass index of 18.0 to 30.0 kg/m^2^ who were able to and agreed to discontinue all iron preparations for 14 days before study drug administration. Participants were excluded if they had a current or past history of cardiovascular, cerebrovascular, pulmonary, renal, or liver disease; a positive test result for hepatitis B or C, or HIV infection; or a current or past history of gastric or duodenal ulcers or other gastrointestinal disease that could interfere with study drug absorption. Other key exclusion criteria included use of chronic daily medication, any prescription medication or over‐the‐counter multivitamin supplements within 14 days, or use of any herbal supplements within 28 days before admission to the study site; or a known history of smoking and/or use of nicotine or nicotine‐containing products within 6 months of screening. Participants were also required to abstain from consumption of alcohol within 48 hours of admission to the study site.

### 1 Safety and Tolerability Assessments

Safety and tolerability were evaluated in both studies through adverse event (AE) monitoring (coded according to Medical Dictionary for Regulatory Activities version 23.0 for study 1 and version 17.1 for study 2), 12‐lead electrocardiogram (ECG) recordings, vital signs, physical examinations, and laboratory tests. Telephone follow‐up with participants was conducted by 14 days after the last dose. The safety population included all participants who received at least 1 dose of vadadustat.

### Pharmacokinetic Sampling

In both studies, blood samples for PK analysis of vadadustat were taken before dosing and at 0.5, 1, 1.5, 2, 3, 4, 6, 12, 16, and 24 hours after vadadustat dosing. Additional samples were taken at 9 and 48 hours after dosing in study 1 and 8 hours after dosing in study 2. Plasma concentrations of vadadustat and its metabolite vadadustat‐O‐glucuronide were determined using validated liquid chromatography with tandem mass spectrometry; additional details have been published previously^19^ and are also presented in Table S2. The lower limit of quantification was 100 ng/mL for vadadustat in both study 1 and study 2 and was 100 and 5.0 ng/mL for vadadustat‐O‐glucuronide in study 1 and study 2, respectively; samples below the limit of quantification were reported as 0.

### Pharmacokinetic Analyses

PK parameters included vadadustat area under the plasma concentration–time curve (AUC) from time 0 to the last quantifiable concentration (AUC_0‐last_), AUC from time 0 to infinity (AUC_0‐∞_), maximum observed plasma concentration (C_max_), time to maximum observed plasma concentration (t_max_), apparent terminal elimination half‐life (t_½_), fraction of AUC extrapolated from time 0 to infinity, and apparent total body clearance (CL/F). PK parameters were also calculated for vadadustat‐O‐glucuronide in study 2.

### Statistical Analyses

In study 1, a sample size of 18 participants for each part of the study was selected to provide 90% power to detect a +50% or –33% difference in vadadustat PK parameters. These parameters were based on a review of vadadustat PK variability finding a coefficient of variation of 30% for vadadustat and a true geometric mean ratio of 1, and allowing for 10% dropout. In study 2, a sample size of 10 participants was selected to provide ≈80% power for confirming that the 90% confidence interval (CI) falls within 80% to 125% based on a review of vadadustat PK variability (intraindividual coefficient of variation in ln‐transformed C_max_ of 17.7%). To assess the effect of coadministration of phosphate binder or ferrous sulfate on vadadustat PK, an analysis of variance (ANOVA) was conducted on ln‐transformed AUC_0‐last_, AUC_0‐∞_, and C_max_. This was done at an alpha level of 0.05, including treatment as a fixed effect and participant as a random effect in study 1, and sequence, participant, period, and treatment as factors in study 2. The ratio of geometric means and corresponding 90%CIs were calculated for vadadustat with and without a phosphate binder using least squares (LS) means from the ANOVA of ln‐transformed AUC_0‐last_, AUC_0‐∞_, and C_max_. Plasma PK parameters were estimated using Phoenix WinNonlin software (version 8.0 in study 1 and version 6.3 in study 2; Pharsight Corporation, Mountain View, California) or SAS software version 9.2 (SAS Institute, Inc., Cary, North Carolina).

## Results

### Participant Disposition and Baseline Characteristics

A total of 54 participants (35 men, 19 women) were enrolled in study 1 (18 participants in each part), and 10 male participants were enrolled in study 2. All participants completed each study per protocol and were included in the PK and safety analyses. Study 1 was conducted from May 19, 2020, to August 2, 2020. Study 2 was conducted from December 2, 2014, to December 20, 2014. The demographics and baseline characteristics of participants were well balanced between treatment groups within each study (Table 1).

**Table 1:** Demographics and Baseline Characteristics

	**Study 1 (N = 54)**	**Study 2 (N = 10)**
Age, y, mean (SD)	38.6 (10.6)	32.5 (7.3)
Sex, n (%)		
Male	35 (64.8)	10 (100.0)
Female	19 (35.2)	0 (0)
Race, n (%)		
White	48 (88.9)	6 (60.0)
Black or African American	5 (9.3)	3 (30.0)
Asian	1 (1.9)	0 (0)
Native Hawaiian or Pacific Islander	0 (0)	1 (10.0)
Ethnicity, n (%)		
Hispanic or Latino	8 (14.8)	0 (0)
Not Hispanic or Latino	46 (85.2)	10 (100.0)
Height, cm, mean (SD)	170.8 (9.2)	173.6 (5.4)
Weight, kg, mean (SD)	74.8 (11.7)	79.4 (12.9)
BMI, kg/m^2^ (SD)	25.6 (2.8)	26.2 (3.1)

BMI, body mass index; SD, standard deviation.

### Effect of Phosphate Binders on the PK of Vadadustat (Study 1)

**Figure 1:**
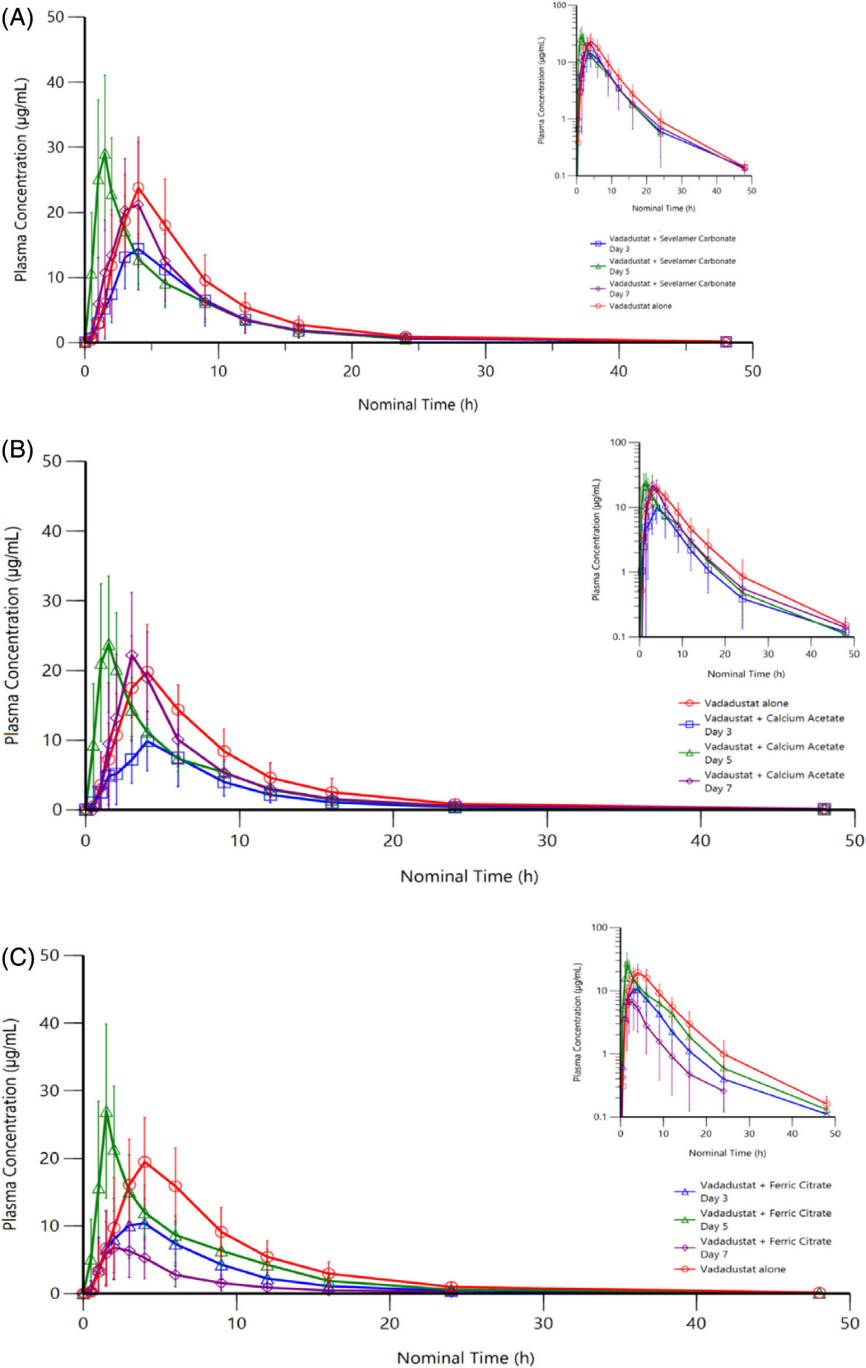
Mean (SD) plasma concentration of vadadustat following coadministration with (A) sevelamer carbonate, (B) calcium acetate, and (C) ferric citrate. Insets represent data in logarithmic scale. SD, standard deviation.

**Figure 2:**
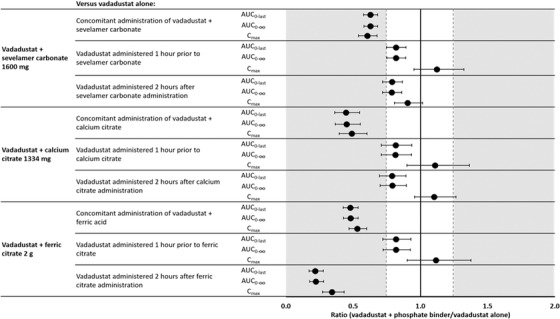
Effect of a single dose of phosphate binder on the PK of a single dose of vadadustat (300 mg). Values shown are geometric least squares mean ratios; the error bars represent the 90% geometric confidence interval. AUC, area under the plasma concentration–time curve; AUC_0‐∞_, area under the plasma concentration–time curve from time 0 to infinity; AUC_0‐last_, area under the plasma concentration–time curve from time 0 to last quantifiable concentration; C_max_, maximum observed plasma concentration; PK, pharmacokinetics.

Vadadustat plasma PK parameter findings are summarized in Table 2. Plasma concentration–time profiles for vadadustat alone and when coadministered with sevelamer carbonate, calcium acetate, and ferric citrate are presented in Figure 1. Concomitant administration of phosphate binders with vadadustat reduced vadadustat AUC_0‐last_, AUC_0‐∞_, and C_max_ compared with administering vadadustat alone, whereas t_max_ and t_½_ remained similar and CL/F increased. ANOVA indicated that concomitant administration of phosphate binders with vadadustat reduced vadadustat AUC_0‐last_ and AUC_0‐∞_ by 37% to 55% and C_max_ by 40% to 51% (Figure 2). Specifically, the geometric LS mean AUC_0‐∞_ for concurrent vadadustat and sevelamer carbonate was 113.98 μg · h/mL, with a geometric LS mean ratio of 62.85% (90%CI, 57.96‐68.16) compared to vadadustat alone. When vadadustat was administered concurrently with calcium acetate, the geometric LS mean AUC_0‐∞_ was relatively lower at 72.44 μg · h/mL, with a geometric LS mean ratio of 45.00% (90%CI, 36.67‐55.24) compared to vadadustat alone. Likewise, the geometric LS mean AUC_0‐∞_ for concurrent vadadustat and ferric citrate was 80.90 μg · h/mL, with a geometric LS mean ratio of 47.93% (90%CI, 42.75‐53.75) compared to vadadustat alone.

**Table 2:** Summary of Plasma PK Parameters for Vadadustat Following Coadministration With Sevelamer Carbonate, Calcium Acetate, or Ferric Citrate

	Sevelamer Carbonate + Vadadustat				Calcium Acetate + Vadadustat				Ferric Citrate + Vadadustat			
PK Parameters	Vadadustat Alone^a^ (n = 18)	Concomitant Administration^b^ (n = 18)	Vadadustat Administered 1 h Before Sevelamer Carbonate^c^ (n = 18)	Vadadustat Administered 2 h After Sevelamer Carbonate^d^ (n = 18)	Vadadustat Alone^a^ (n = 18)	Concomitant Administration^b^ (n = 18)	Vadadustat Administered 1 h Before Calcium Acetate^c^ (n = 17)	Vadadustat Administered 2 h After Calcium Acetate^d^ (n = 18)	Vadadustat Alone^a^ (n = 18)	Concomitant Administration^b^ (n = 18)	Vadadustat Administered 1 h Before Ferric Citrate^c^ (n = 17)	Vadadustat Administered 2 h After Ferric Citrate^d^ (n = 18)
AUC_0‐last_, μg·h/mL	185 ± 57.3	121 ± 50.4	152 ± 46.2	151 ± 57.5	163 ± 45.7	77.4 ± 31.8	130 ± 36.2	132 ± 44.3	173 ± 56.6	85.3 ± 38.6	143 ± 43.3	42.7 ± 24.5
AUC_0‐∞_, μg·h/mL	190 ± 57.2	124 ± 51.0	156 ± 48.5	154 ± 57.8	166 ± 45.4	79.6 ± 32.7	133 ± 36.8	135 ± 44.3	176 ± 56.4	87.5 ± 38.8	147 ± 43.8	44.3 ± 25.3
C_max_, μg/mL	26.7 ± 6.6	16.6 ± 5.8	31.3 ± 10.7	24.6 ± 7.6	21.9 ± 5.6	11.3 ± 4.4	25.0 ± 9.6	24.6 ± 7.1	22.3 ± 5.9	12.1 ± 4.3	27.3 ± 12.6	8.58 ± 4.4
t_max_, h	4.0 (2.9, 6.0)	3.5 (2.0, 6.1)	1.5 (0.9, 6.0)	3.0 (0.9, 5.9)	3.9 (1.4, 6.0)	4.0 (1.4, 6.0)	1.4 (0.9, 4.0)	3.0 (1.5, 4.0)	3.9 (1.4, 5.9)	3.0 (1.5, 6.0)	1.4 (1.0, 6.0)	2.0 (1.4, 3.9)
Elimination half‐life, h	4.8 ± 1.2	4.7 ± 0.9	4.5 ± 0.7	5.1 ± 1.0	4.8 ± 1.20	4.6 ± 0.90	4.5 ± 0.8	4.8 ± 1.0	5.3 ± 1.4	4.6 ± 0.9	4.3 ± 0.9	4.8 ± 1.2
CL/F, L/h	1.7 ± 0.6	2.9 ± 1.4	2.1 ± 0.8	2.3 ± 1.1	1.9 ± 0.4	4.6 ± 2.5	2.4 ± 0.6	2.5 ± 1.1	1.8 ± 0.5	4.0 ± 1.6	2.2 ± 0.7	9.8 ± 7.1

AUC_0‐∞_, area under the plasma concentration–time curve from dosing (time 0) to infinity; AUC_0‐last_, area under the plasma concentration–time curve from dosing (time 0) to last quantifiable concentration; CL/F, apparent total body clearance; C_max_, maximum observed plasma concentration; PK, pharmacokinetics; SD, standard deviation; t_max_, time to maximum observed plasma concentration.

Values are represented as mean ± SD, except for t_max_, which is presented as median (minimum, maximum).

^a^ Vadadustat 300 mg administered immediately after breakfast.

^b^ Vadadustat 300 mg administered concomitantly with phosphate binder (sevelamer carbonate 1600 mg/calcium acetate 1334 mg/ferric citrate 2 g) immediately after breakfast.

^c^ Vadadustat 300 mg administered under fasting conditions and phosphate binder (sevelamer carbonate 1600 mg/calcium acetate 1334 mg/ferric citrate 2 g) administered 1 hour later, followed immediately (within 2 minutes) by breakfast.

^d^ Phosphate binder (sevelamer carbonate 1600 mg/calcium acetate 1334 mg/ferric citrate 2 g) followed immediately by breakfast; vadadustat 300 mg administered 2 hours later.

Administration of vadadustat 2 hours after ferric citrate resulted in a greater reduction in vadadustat exposure compared with concomitant administration, which was not observed with the other phosphate binders. Specifically, ferric citrate reduced vadadustat AUC_0‐last_ and AUC_0‐∞_ by 78% and C_max_ by 66% (Figure 1B). The geometric LS mean AUC_0‐∞_ for vadadustat 2 hours after ferric citrate was 37.47 μg · h/mL, with a geometric LS mean ratio of 22.20% (90%CI, 17.54‐28.10) compared to vadadustat alone. However, when vadadustat was administered 1 hour before ferric citrate, we observed a smaller 18% reduction in AUC_0‐last_ and AUC_0‐∞_ and an 11% increase in C_max_. Specifically, the geometric LS mean AUC_0‐∞_ for vadadustat 1 hour before ferric citrate was 138.00 μg · h/mL, with a geometric LS mean ratio of 81.76% (90%CI, 72.23‐92.55) compared to vadadustat alone.

### Effect of Ferrous Sulfate Coadministration on the PK of Vadadustat (Study 2)

**Figure 3:**
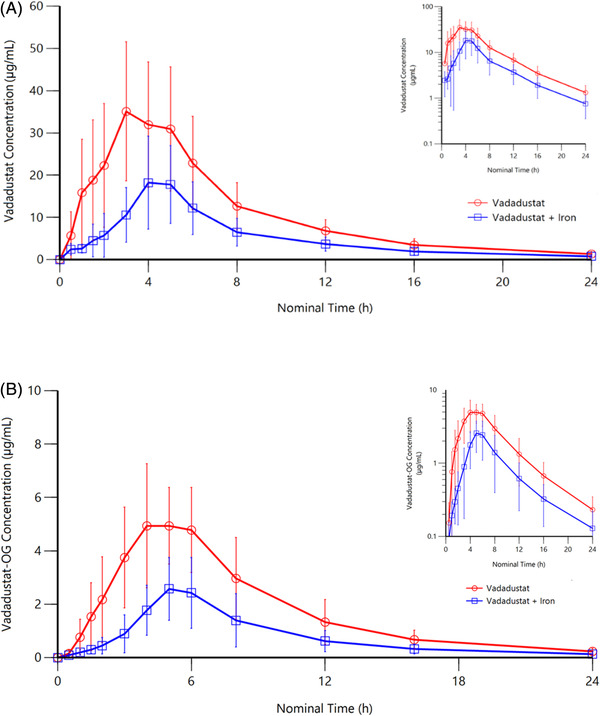
Mean (SD) plasma concentration of (A) vadadustat and (B) vadadustat‐O‐glucuronide following administration of vadadustat 450 mg with or without ferrous sulfate 325 mg (equivalent to 65 mg of elemental iron). Insets represent data in logarithmic scale. OG, O‐glucuronide; SD, standard deviation.

Vadadustat exposure was reduced by concurrent administration of ferrous sulfate, with AUC_0‐last_ and AUC_0‐∞_ reduced by 54% and C_max_ by 51% (Table 3). The geometric LS mean AUC_0‐∞_ for concurrent vadadustat and ferrous sulfate was 122 μg · h/mL, with a geometric LS mean ratio of 46.3 μg · h/mL (90%CI, 37.1‐57.8) compared to vadadustat alone. Median t_max_ was extended to 4 hours when vadadustat was coadministered with ferrous sulfate compared with 3 hours when vadadustat was administered alone, whereas the t_½_ remained similar at ≈5 hours. Mean plasma vadadustat concentrations exhibited a monoexponential decline after C_max_ was reached (Figure 3). Although mean CL/F increased by ≈20% when vadadustat was coadministered with ferrous sulfate, this was attributed to the lower AUC estimates rather than changes in vadadustat elimination. Changes in plasma vadadustat‐O‐glucuronide PK reflected changes in the parent drug, with AUC_0‐last_, AUC_0‐∞_, and C_max_ significantly reduced by ≥50% when vadadustat was coadministered with ferrous sulfate.

**Table 3:** Summary of Plasma PK Parameters for Vadadustat and Vadadustat‐O‐Glucuronide Following Administration of Vadadustat 450 mg With or Without Ferrous Sulfate 325 mg (Equivalent to 65 mg of Elemental Iron)

	**Vadadustat**		**Vadadustat‐O‐Glucuronide**	
**PK Parameters**	**Vadadustat (n = 10)**	**Vadadustat + Ferrous Sulfate (n = 10)**	**Vadadustat (n = 10)**	**Vadadustat + Ferrous Sulfate (n = 10)**
AUC_0‐last_, μg · h/mL	260 ± 59.1	123 ± 39.6	42.8 ± 10.8	18.6 ± 8.2
AUC_0‐∞_, μg · h/mL	271 ± 61.5	128 ± 42.2	44.5 ± 11.4	19.7 ± 9.0
%AUC_0‐extrap_	3.7 ± 1.7	4.4 ± 2.2	3.8 ± 1.7	5.3 ± 3.1
C_max_, μg/mL	46.6 ± 11.1	23.7 ± 8.4	6.07 ± 1.2	2.8 ± 1.1
t_max_, h	3.0 (1.5, 5.0)	4.0 (1.5, 5.0)	4.0 (3.0, 6.0)	5.0 (3.0, 6.0)
t_½_, h	5.2 ± 0.8	5.1 ± 1.0	5.1 ± 0.9	5.5 ± 1.3
CL/F, L/h	1.8 ± 0.5	3.9 ± 1.5	NA	NA
GMR AUC_0‐last_	46.0 (36.7‐57.6)		NC	
GMR AUC_0‐∞_	46.3 (37.1‐57.8)		NC	
GMR C_max_	49.3 (37.8‐64.4)		NC	

AUC_0‐∞_, area under the plasma concentration–time curve from time 0 to infinity; AUC_0‐last_, area under the plasma concentration–time curve from dosing time 0 to the last quantifiable concentration; %AUC_0‐extrap,_ fraction of area under the plasma concentration–time curve extrapolated from time 0 to infinity; CL/F; apparent total body clearance; C_max_, maximum observed plasma concentration; GMR, geometric mean ratio; NA, not applicable; NC, not calculated; PK, pharmacokinetics; SD, standard deviation; t_max_, time to maximum observed plasma concentration.

Values are presented as mean ± SD, except for t_max_, which is presented as median (minimum, maximum), and GM ratios (vadadustat + ferrous sulfate/vadadustat), which are presented as percentages (90% confidence interval).

### Safety

Vadadustat was generally well tolerated when coadministered with sevelamer carbonate, calcium acetate, or ferric citrate (Table S3). No AEs were reported when ferrous sulfate was coadministered with vadadustat.

In the sevelamer carbonate cohort, a total of 35 treatment‐emergent AEs (TEAEs) were recorded for 13 (72%) participants, of which 24 were considered to be related to the study drug. Seven treatment‐related TEAEs were recorded for 5 (28%) participants administered vadadustat alone vs 17 treatment‐related TEAEs among 6 (33%) participants administered vadadustat and sevelamer carbonate.

In the calcium acetate cohort, a total of 36 TEAEs were reported in 14 (78%) participants, including 23 treatment‐related TEAEs. Nine treatment‐related TEAEs were recorded for 7 (39%) participants administered vadadustat alone vs 14 treatment‐related TEAEs among 8 (44%) participants administered vadadustat and calcium acetate.

In the ferric citrate cohort, 37 TEAEs were reported in 9 (50%) participants overall, including 29 treatment‐related TEAEs. Nine treatment‐related TEAEs were recorded for 5 (28%) participants administered vadadustat alone vs 20 treatment‐related TEAEs among 7 (39%) participants administered vadadustat and ferric citrate.

The most common vadadustat‐related TEAEs were headache, nausea, constipation, and hot flush, whereas the most frequent phosphate binder–related TEAEs were headache, nausea, and hot flush. However, all TEAEs were mild in severity; no moderate or severe AEs were reported in >2 participants in any treatment group. Furthermore, no deaths, serious AEs, or TEAEs leading to discontinuation of the study drug were reported, and there were no clinically significant findings relating to laboratory tests, vital signs, or ECGs in either study.

## Discussion

Hyperphosphatemia and anemia are common complications of CKD. Phosphate binders and iron supplements may be administered concurrently to patients receiving vadadustat. Notably, phosphate binders and iron supplements have been shown to interfere with the absorption of numerous drugs, including immunosuppressants, antiplatelet agents, and antibiotics,^20^, ^21^ and in some cases, coadministration with phosphate binders can increase the risk of drug toxicity.^20^ Therefore, medications are often administered 1 to 2 hours before or 3 to 6 hours after a phosphate binder to avoid DDIs.^20^ Here, we report the results of 2 studies evaluating the effect of different classes of phosphate binders and supplemental iron on the PK, safety, and tolerability of a single oral dose of vadadustat in healthy adults.

In both studies, a potential DDI was observed when vadadustat was coadministered with the phosphate binders sevelamer carbonate, calcium acetate, ferric citrate, or ferrous sulfate. DDI with non–iron phosphate binders can be overcome by administering vadadustat immediately upon waking, before breakfast, or 2 hours after administering a phosphate binder (which can continue to be administered with meals). DDI with ferric citrate can be reduced by administering vadadustat under fasting conditions before an iron‐containing phosphate binder. The median t_½_ of vadadustat remained unchanged by phosphate binders, irrespective of class of phosphate binder or the relative staggering of vadadustat and phosphate‐binder dosing. This implies that vadadustat absorption, rather than elimination, was affected.

The observations in participants administered ferric citrate and vadadustat may reflect the unique properties of the branded ferric citrate used in the current study. The branded ferric citrate has a larger surface area than nonpharmaceutical, commercial‐grade ferric citrate,^22^ and a phosphate‐binding capacity that is greater than that of calcium carbonate but comparable to calcium acetate.^23^ Taken together, there may exist a potential for substantial vadadustat–ferric citrate complex formation with delayed absorption even when vadadustat is administered 2 hours after ferric citrate.

The bioavailability of vadadustat was also significantly reduced when it was administered concurrently with ferrous sulfate. Furthermore, PK parameters for the metabolite vadadustat‐O‐glucuronide were similarly reduced by concurrent administration, while metabolite‐to‐parent drug ratios for AUC and C_max_ remained comparable, indicating that vadadustat metabolism was not affected by coadministration.

The potential for DDIs between HIF‐PHIs and phosphate binders has been previously reported. In healthy individuals, roxadustat exposure was markedly reduced by 67% and 46% when administered concomitantly with sevelamer carbonate or calcium acetate, respectively.^24^ Time‐separated roxadustat dosing 1 hour before or 1 hour following phosphate binder administration was sufficient to overcome the DDI.^24^ Similarly, coadministration of molidustat and ferrous sulfate reduced molidustat AUC by 75% and 51% under fasted and fed conditions, respectively, whereas administration of molidustat 2 hours after ferrous sulfate reduced molidustat AUC by 16%.^25^

Overall, vadadustat was well tolerated when coadministered with phosphate binders. There were no deaths, serious TEAEs, or TEAEs leading to discontinuation, and no clinically significant changes in laboratory tests, vital signs, or ECGs were observed. Furthermore, all TEAEs were mild in severity, and vadadustat‐related AEs were consistent with those reported previously.^13^, ^16^, ^17^, ^26^ Vadadustat was similarly well tolerated when coadministered with ferrous sulfate, with no AEs reported.

The current studies evaluated only a single dose of vadadustat, phosphate binder, or ferrous sulfate over a short duration of treatment and in healthy participants only. Therefore, the influence of repeat dosing on vadadustat PK, safety, and tolerability was not determined.

## Conclusion

Coadministration of a single oral dose of vadadustat with a phosphate binder or iron supplement reduces vadadustat exposure; however, the impact of these DDIs can be reduced by administering vadadustat 1 hour before phosphate binders or oral ferrous sulfate. Furthermore, vadadustat was well tolerated when administered in conjunction with phosphate binders or oral ferrous sulfate.

## Funding

This study was funded by Akebia Therapeutics, Inc. Medical writing assistance was provided by Cadent Medical Communications, LLC, a Syneos Health group company, and was supported by Akebia Therapeutics, Inc.

## Conflicts of Interest

S.K.P., J.M., R.S., S.K.B., and A.C. are employees of Akebia Therapeutics, Inc.

## Author Contributions

All authors provided substantial contributions to the conception of the work. All authors substantially contributed to the acquisition, analysis, or interpretation of data for the manuscript. All authors participated in drafting, revising, and critically reviewing the manuscript for important intellectual content. All authors approved the final version of this manuscript to be published and agree to be accountable for all aspects of the work in ensuring that questions related to the accuracy or integrity of any part of the work are appropriately investigated and resolved.

## Data‐Sharing Statement

Data sharing requests can be submitted to medicalinfo@akebia.com and will be reviewed by an independent review board, with final approval by Akebia. All approved proposals are subject to a research agreement, and data will be made available in a third‐party vendor software.

## Supporting information

SUPPLEMENTARY INFORMATION

SUPPLEMENTARY INFORMATION

SUPPLEMENTARY INFORMATION
