# Supplementary material for: Effect of Phosphate Binders and a Dietary Iron Supplement on the Pharmacokinetics of a Single Dose of Vadadustat in Healthy Adults
Source: Clin Pharmacol Drug Dev. 2022 Feb 16;11(4):475–85. doi: 10.1002/cpdd.1033 (PMC9305443; doi:10.1002/cpdd.1033)
Supplement: Supplementary file 1 — SUPPLEMENTARY INFORMATION [file CPDD-11-475-s002.docx]

**Table S1.** Inclusion and Exclusion Criteria (Study 1)

| **Inclusion criteria** | **Exclusion criteria** |
| --- | --- |
| To be considered eligible, all of the following criteria must have been met:   1. Male or female participants, aged 18–55 years, inclusive, at time of informed consent    1. Female participants of childbearing potential who were not lactating and not pregnant, as confirmed by a negative serum pregnancy test at screening and Day –1, and using, and agreed to continue using, an effective method of contraception for at least 4 weeks prior to the first dose of study drug until 30 days after the last dose of study drug    2. Female participants of non-childbearing potential who were surgically sterile (eg, hysterectomy, bilateral tubal ligation, oophorectomy) or postmenopausal (no menses for >1 year with follicle-stimulating hormone level >40 U/L at screening)    3. Female participants of childbearing potential who agreed to not donate ova during the study and for at least 30 days after the last dose of study drug    4. Male participants who had not had a vasectomy in at least 6 months and agreed to use an effective method of contraception during the study and until 90 days after the last dose of the study drug, and to not donate sperm during the study and for at least 90 days after the last dose of study drug 2. Healthy per investigator judgment as documented by medical history, physical examination, vital sign assessments, 12-lead electrocardiogram (ECG), clinical laboratory assessments, and general observations    1. At screening, abnormalities or deviations outside the normal ranges for any clinical assessments (laboratory tests, ECG, vital signs) may have been repeated once at the discretion of the investigator(s), and results that continued to be outside the normal ranges must be judged by the investigator to be not clinically significant and acceptable for study participation    2. On Day –1, alanine aminotransferase, aspartate aminotransferase, and total bilirubin values must have been within the upper limits of the normal range. All other laboratory test results that were outside the normal range on Day –1 and judged by the investigator to be not clinically significant may have been repeated. Results that continued to be outside the normal range must have been judged by the investigator to be not clinically significant and acceptable for study participation 3. Body mass index between 18.0 and 30.0 kg/m^2^, with a minimum body weight of 45.0 kg for women and 50.0 kg for men, inclusive 4. Understood the procedures and requirements of the study and provided written informed consent and authorization for protected health information disclosure 5. Willing and able to comply with the requirements of the study protocol | Participants who met any of the following criteria were ineligible for participation in the study:   1. Current or past history of cardiovascular, cerebrovascular, respiratory, gastrointestinal, hematologic, renal, hepatic, immunologic, metabolic, urologic, neurologic, dermatologic, psychiatric, or other major disease, as determined by the investigator. History of cancer (except treated nonmelanoma skin cancer) or history of chemotherapy within 5 years prior to screening 2. Any surgical or medical condition or history that, in the opinion of the investigator, may have potentially altered the absorption, metabolism, or excretion of study treatment, such as, but not limited to, gastric bypass surgery or gastric or duodenal ulcers 3. Clinically significant history of dysphagia, bowel obstruction, or perforation 4. Clinically significant history of hypercalcemia 5. Clinically significant history of iron overload 6. Clinically significant history of liver disease 7. Clinically significant history of hypophosphatemia, ulcerative colitis, or gastrointestinal bleeding 8. Contraindication to study drugs or their excipients and/or history of allergic or anaphylactic reactions    1. Taken any of the following prohibited medications: 9. Any prescription medication or over-the-counter multivitamin supplement, or any nonprescription products (including herbal containing preparations but excluding acetaminophen up to 2 g daily) within 14 days prior to Day –1 10. Any drug known to inhibit or induce cytochrome P450 (CYP) enzymes and/or P glycoprotein, including St. John’s wort (*Hypericum perforatum*) within 14 days or 5 half-lives (whichever is longer) prior to Day –1 11. History of drug abuse within the previous year prior to screening or use of soft drugs (such as marijuana) within 3 months prior to the screening visit or hard drugs (such as cocaine, phencyclidine [PCP], crack, opioid derivatives including heroin, and amphetamine derivatives) within 1 year prior to screening 12. History of regular alcohol consumption exceeding 14 drinks/week (1 drink = 5 ounces [150 mL] of wine or 12 ounces [360 mL] of beer or 1.5 ounces [45 mL] of hard liquor) within 6 months of screening or alcohol abuse within 1 year prior to screening 13. Positive drug and alcohol test at screening or on  Day –1 14. History of latent or active tuberculosis as per documented medical history. Exposure to endemic areas within 8 weeks of screening 15. Daily use of nicotine-containing products within 6 months of screening 16. Consumed any food or drink/beverage containing grapefruit or grapefruit juice, apple or orange juice, pomelo juice, pomegranate, pineapple, star fruit, Seville or Moro (blood) orange products, and vegetables from the mustard green family (eg, kale, broccoli, watercress, collard greens, kohlrabi, Brussels sprouts, mustard greens) and charbroiled meats known to modulate CYP enzyme activity and transporters within 7 days before administration of study drug 17. Positive test results of hepatitis B surface antigen, or positive hepatitis C virus antibody test result within 3 months prior to Day –1 or at screening 18. Positive test results for human immunodeficiency virus antibody within 3 months prior to Day –1 or at screening 19. Participation in another clinical study or exposure to any investigational agent within 30 days or 5 half-lives prior to Day –1, whichever was longer 20. Donation of plasma within 7 days prior to dosing. Donation or loss of blood (excluding volume drawn at screening) of 50 mL to 499 mL of blood within 30 days, or more than 499 mL within 56 days prior to the first dosing 21. Received a tattoo or body piercing (including ear piercings) within 2 months prior to Day 1, and/or open wound that may result in risk of infection 22. Had a condition that the investigator believed would interfere with his/her ability to provide written informed consent, comply with study instructions, might confound the interpretation of the study results, or put the participant at undue risk 23. Have previously participated in a clinical study that administered vadadustat |
