# Supplementary material for: Effect of Phosphate Binders and a Dietary Iron Supplement on the Pharmacokinetics of a Single Dose of Vadadustat in Healthy Adults
Source: Clin Pharmacol Drug Dev. 2022 Feb 16;11(4):475–85. doi: 10.1002/cpdd.1033 (PMC9305443; doi:10.1002/cpdd.1033)
Supplement: Supplementary file 2 — SUPPLEMENTARY INFORMATION [file CPDD-11-475-s001.docx]

**Table S2.** Pharmacokinetic Sampling and Analytical Methods

| **Study 1** | **Study 2** |
| --- | --- |
| - Plasma concentrations of vadadustat and vadadustat-O-glucuronide were measured at Syneos Health (Quebec, Canada) using a validated liquid chromatography (HPLC) with tandem mass spectrometry (MS/MS) method - In brief, internal standards were added to plasma samples. Samples were then diluted with 2% phosphoric acid in water, vortexed, and centrifuged. After centrifugation, the samples were extracted by solid phase extraction and eluted using methanol/ acetonitrile (50/50) containing 1% formic acid - The eluant, containing vadadustat, vadadustat-O-glucuronide, and the internal standards, was then analyzed using an HPLC-MS/MS system. The HPLC separation was performed using a C18 column, and detection was carried out by an MS/MS detector equipped with a Turbo Spray ion source - For each sample, the ratio between the peak area of vadadustat and vadadustat-O-glucuronide and their internal standards and the calibration curve equation were used to calculate the concentration of vadadustat and vadadustat-O-glucuronide - When using 0.05 mL of plasma sample, the lower limit of quantification was 100 ng/mL for both vadadustat and vadadustat-O-glucuronide | - Vadadustat, vadadustat-acyl-glucuronide, and vadadustat-O-glucuronide and IS (d_3_‑vadadustat and alprazolam) were extracted from 150 µL of human K2-EDTA plasma treated with 2% glacial acetic acid (GAA) using 96-well Waters^®^ HLB plates (Waters, Wexford, Ireland) - After elution from the extraction plate with 1% formic acid in 50:50 (v/v) ACN:MeOH, the extracts containing the analytes and IS were injected onto a Waters Acquity^®^ BEH C18 (2.1 × 50 mm, 1.7 µm) column - The peak areas of analytes and IS were detected using positive ion TurboIonSpray^®^ mode - Quantitation was based on quadratic regression analysis (with 1/x^2^ weighting) of the calibration curve obtained using the area ratio of the peaks related to the 307→232 mass transition for vadadustat, the 312→237 IS mass transition for d_3_-vadadustat versus theoretical concentrations of the standards, and the area ratio of the peaks related to the mass transitions of 483→307 for vadadustat-acyl-glucuronide and 483→307 for vadadustat-O-glucuronide, both relative to the 309→281 IS mass transition for alprazolam versus theoretical concentrations of the standards |
