# Supplementary material for: Effect of Phosphate Binders and a Dietary Iron Supplement on the Pharmacokinetics of a Single Dose of Vadadustat in Healthy Adults
Source: Clin Pharmacol Drug Dev. 2022 Feb 16;11(4):475–85. doi: 10.1002/cpdd.1033 (PMC9305443; doi:10.1002/cpdd.1033)
Supplement: Supplementary file 3 — SUPPLEMENTARY INFORMATION [file CPDD-11-475-s003.docx]

**Table S3.** Summary of TEAEs

|  | **Vadadustat + sevelamer carbonate** | | | | | | **Vadadustat + calcium acetate** | | | | | | **Vadadustat + ferric citrate** | | | | | |
| --- | --- | --- | --- | --- | --- | --- | --- | --- | --- | --- | --- | --- | --- | --- | --- | --- | --- | --- |
|  | **Vadadustat**  **(n=18)** | | | **Vadadustat + sevelamer carbonate**  **(n=18)** | | | **Vadadustat**  **(n=18)** | | | **Vadadustat + calcium acetate**  **(n=18)** | | | **Vadadustat**  **(n=18)** | | | **Vadadustat + ferric citrate**  **(n=18)** | | |
|  | Events, n | Participants, n (%) | | Events, n | Participants, n (%) | | Events, n | Participants, n (%) | | Events, n | Participants, n (%) | | Events, n | Participants, n (%) | | Events, n | Participants, n (%) | |
| TEAEs | 10 | 7 (38.9) | | 25 | 10 (55.6) | | 11 | 8 (44.4) | | 25 | 12 (66.7) | | 13 | 8 (44.4) | | 24 | 7 (38.9) | |
| Treatment-related AEs | 7 | 5 (27.8) | | 17 | 6 (33.3) | | 9 | 7 (38.9) | | 14 | 8 (44.4) | | 9 | 5 (27.8) | | 20 | 7 (38.9) | |
| Treatment-related TEAEs observed in ≥2 (11%) participants | | | | | | | | | | | | | | | | | | |
| Headache  Vadadustat-related  Phosphate binder–related | 3  – | | 3 (16.7)  – | 2  2 | | 1 (5.6)  1 (5.6) | 2  – | | 2 (11.1)  – | 4  5 | | 3 (16.7)  4 (22.2) | 1  – | | 1 (5.6)  – | 2  2 | | 2 (11.1)  2 (11.1) |
| Somnolence  Vadadustat-related  Phosphate binder–related | 1  – | | 1 (5.6)  – | 3  3 | | 2 (11.1)  2 (11.1) | 0  – | | 0  – | 0  0 | | 0  0 | 0  – | | 0  – | 0  0 | | 0  0 |
| Constipation  Vadadustat-related  Phosphate binder–related | 2  – | | 2 (11.1)  – | 0  0 | | 0  0 | 1  – | | 1 (5.6)  – | 0  0 | | 0  0 | 1  – | | 1 (5.6)  – | 0  0 | | 0  0 |
| Bradyphrenia  Vadadustat-related  Phosphate binder–related | 0  – | | 0  – | 2  2 | | 2 (11.1)  2 (11.1) | 0  – | | 0  – | 0  0 | | 0  0 | 0  – | | 0  – | 0  0 | | 0  0 |
| Hot flush  Vadadustat-related  Phosphate binder–related | 0  – | | 0  – | 2  2 | | 2 (11.1)  2 (11.1) | 0  – | | 0  – | 0  0 | | 0  0 | 0  – | | 0  – | 2  2 | | 2 (11.1)  2 (11.1) |
| Abdominal distention  Vadadustat-related  Phosphate binder–related | 0  – | | 0  – | 0  0 | | 0  0 | 0  – | | 0  – | 1  1 | | 1 (5.6)  1 (5.6) | 2  – | | 2 (11.1)  – | 0  1 | | 0  1 (5.6) |
| Nausea  Vadadustat-related  Phosphate binder–related | 1  – | | 1 (5.6)  – | 1  0 | | 1 (5.6)  0 | 1  – | | 1 (5.6)  – | 2  3 | | 1 (5.6)  2 (11.1) | 0  – | | 0  – | 2  2 | | 2 (11.1)  2 (11.1) |
| Fatigue  Vadadustat-related  Phosphate binder–related | 0  – | | 0  – | 0  0 | | 0  0 | 0  – | | 0  – | 2  0 | | 2 (11.1)  0 | 0  – | | 0  – | 0  0 | | 0  0 |
| ALT increased  Vadadustat-related  Phosphate binder–related | 0  0 | | 0  0 | 2  2 | | 2 (11.1)  2 (11.1) | 0  0 | | 0  0 | 0  0 | | 0  0 | 0  0 | | 0  0 | 0  0 | | 0  0 |
| Diarrhea  Vadadustat-related  Phosphate binder–related | 0  0 | | 0  0 | 0  0 | | 0  0 | 0  0 | | 0  0 | 0  0 | | 0  0 | 1  0 | | 1 (5.6)  0 | 4  4 | | 2 (11.1)  2 (11.1) |

AE, adverse event; ALT, alanine aminotransferase; TEAE, treatment-emergent adverse event.
